# Nothing about us, without us? A reflection on and call for involving children in the process of valuing child health

**DOI:** 10.1007/s10198-025-01823-1

**Published:** 2025-07-30

**Authors:** Ava Hoogenboom, Vivian Reckers-Droog, Stefan Lipman, Werner Brouwer

**Affiliations:** 1 Erasmus School of Health Policy & Management, Erasmus University Rotterdam, Rotterdam, the Netherlands; 2 Erasmus Centre for Health Economics Rotterdam (EsCHER), Erasmus School of Health Policy and Management (ESHPM), Erasmus University, P.O. Box 1738, Rotterdam, 3000 DR The Netherlands

**Keywords:** Child health state valuation, Children’s involvement, Health-related quality of life, Child health

## Abstract

A key question in economic evaluation is whose preferences health-related quality of life (HRQoL) values should be based on. This question becomes increasingly prominent and complex when it comes to the evaluation of health interventions aimed at children, as this requires the valuation of child health states. Resulting discussions have focused on whose preferences should be elicited, from what perspective, and how – highlighting important challenges. As opposed to current EQ-5D-Y protocol (i.e., valuation of child health states by adult members of the public), this paper explores the potential involvement of children in the process of valuing child health. We identified arguments for public involvement in healthcare decision-making in the broader literature and examined their relevance to involving children in the health state valuation (HSV) process. Overall, in line with recent empirical findings, the arguments provide a basis for broadly exploring the involvement of children. Given the concerns regarding the direct involvement of children in HSV tasks, we call for a shift of thinking in two ways: children can be involved in the process of coming to HRQoL values in more ways than only HSV tasks, and the focus should move from *whether* children should be involved in HSV to exploring *how* they can be involved.

## Valuing child health in cost-utility analyses

Cost-utility analyses (CUAs) are increasingly used to inform reimbursement decisions on (new) health interventions [1], including those (exclusively or also) aimed at children. The calculation of the incremental costs and, especially, the incremental health-related quality of life (HRQoL) gains of new interventions relative to some appropriate comparator, require important normative choices. Commonly, Quality-Adjusted Life-Years (QALYs), comprising both length and quality of life, are used as an outcome measure in CUAs. Anchored on perfect health (1) and dead (0), the relative HRQoL values of different states, which allow QALY calculations, are based on preferences of the group considered most relevant. These preferences are elicited by means of health state valuation (HSV) tasks, like Time Trade-Off (TTO) or Discrete Choice Experiments (DCEs), or a combination of techniques. For the legitimacy and optimality of reimbursement decisions it is crucial to ensure normatively and empirically appropriate elicitation of these preferences. As such, a key question that arises is *whose* preferences should be elicited. Although it also has been suggested to obtain health state preferences in patients who actually experience or have experienced being in the valued health states [2], commonly a representative sample of the adult population (not necessarily experiencing or having experienced the valued health states) is seen as the relevant source [3].

When it comes to evaluating health interventions (also) aimed at children, the question of whose preferences should be elicited for valuing child health states becomes even more prominent—and more complex [4]. Following the increased use of CUAs to inform reimbursement decisions on interventions aimed at children [5], the need for HRQoL instruments that enable the measurement and valuation of child health also increased. The EQ-5D-Y (an adaptation of the adult EQ-5D for use with youth) is a prominent example of such an instrument for children aged 8–15 years [6]. Similar to the recent valuation protocols for the common EQ-5D [7], the current EQ-5D-Y-3 L protocol states that preferences for the related health states should be elicited from *adult* members of the public by means of HSV tasks. Respondents are instructed to take what is often referred to as the ‘child perspective’: “Considering your views about a 10-year-old child, what [health state] do you prefer?” [6]. Following the protocol, children themselves are not asked about their preferences nor involved otherwise [6]. In the adult counterpart (EQ-5D), respondents are asked to consider their own or ‘adult perspective’ [7].

Available evidence suggests that adults attach different HRQoL values to EQ-5D-Y health states compared to similar EQ-5D health states [8–11]. These findings have sparked discussions, mostly among scholars in the field of HSV, about the validity and accuracy of the child HRQoL values based on preferences of adults. As also indicated by Devlin et al. [12], in absence of a golden standard, it remains unclear whether HRQoL values for adult and child health deviate in a justifiable way (e.g., reflecting true differences in preferences, reflecting ‘adjusted preferences’ by perhaps justifiably yet paternalistically overruling expected myopic preferences of children, or to reflect ‘societal’ preferences for child HRQoL).

The discussions have strongly focused on whose preferences should be elicited, from what perspective, and how, with an emphasis on the question of whether to involve children in HSV *tasks* [4, 13]. Arguments for and against doing so have been addressed [13], highlighting important challenges and research gaps. While a few studies [14, 15] have investigated how children could value health states, the arguments against involving children in HSV tasks directly seem to have prevailed given that in current valuation protocols for child health children themselves (and their preferences) do not play any role. This paper, drawing on broader literature of public involvement in healthcare decision-making, advocates for a shift in thinking in two ways. First, we advocate that children can be involved in the *process* of coming to HRQoL values for EQ-5D-Y states in more ways than only through performing HSV tasks. We will refer to this as involving children in the HSV *process*, to distinguish it from the narrower concept of involving children in HSV *tasks*. Second, we advocate to move from asking *whether* children should be involved in the HSV process to exploring *how* they can be involved.

## Involving children in health state valuation

The authors of the EQ-5D-Y-3 L valuation protocol list three arguments for exclusively eliciting preferences from adults in coming to HRQoL values for health states of children [6]. Firstly, they argue that ‘in the same way that countries do not allow people under the age of 18 years to vote which political party should govern the country, it may be viewed as inappropriate to allow a population in the age range of 8–15 years to decide about the population health’ [6]. Secondly, they argue that ‘the valuation task must include consideration of dead to be able to calculate QALYs’ and that ‘it could be considered ethically inappropriate to apply those tasks to a young population of respondents’, and that ‘obtained values that could be inaccurate owing to a possible misunderstanding of the tasks’ [6]. Thirdly, they argue that the ‘taxpayer perspective is in line with what other child health valuation researchers have done’ and that ‘it could be considered fair as it is taxpayers who fund healthcare’ [6].

Not all of these arguments are equally convincing or supported by the general public [17], nor in themselves sufficient to *not* involve children in any way in the HSV process. Apart from the fact that also voting age is subject of discussion and to changes [16], children could be involved in the HSV process in a way that avoids elements like the consideration of dead. Moreover, adults funding healthcare may still care about the actual QALY gains based on children’s preferences, similar to patient values [2].

Looking at the involvement of children in broader health(care) decision-making processes, health authorities increasingly call for and facilitate the involvement of children [18–23]. For example, the National Health Service (NHS) in England has implemented the Youth Advisory Network, where policymakers and service providers seek the opinions of children aged 11 and up on services, resources, and projects they are working on [24]. Moreover, many countries have already established legal frameworks explicating under which circumstances and from what age children should (also) have a say in decisions regarding their health [25–27], which is sometimes seen as a human right that children are entitled to [28]. Several countries allow or even require children from a very young age to be involved in decisions that involve difficult trade-offs affecting their own health(care) [29]. Such a formalisation of children’s involvement in decisions on health(care) demonstrates the value attached by society to children’s views. While HSV is in many ways different and for instance involves hypothetical trade-offs, also of children not forced by adverse circumstances such as severe illness to be confronted with difficult choices and trade-offs in real life, they still may influence decisions affecting the health of actual children. Hence, these broader legal frameworks may provide an indication of the desirability of involving children in the HSV process.

Thinking broadly about children’s involvement, there are convincing arguments to (at least attempt to) *somehow* involve children in the HSV process as this would be in line with the broader trend of involving members of the public and, more specifically, of involving children in (policy) decisions that ‘affect their lives’ [30]. Involving children in the process of HSV does not necessarily entail eliciting their preferences (in some form) by means of (commonly applied) HSV tasks such as TTOs. We do note that recent research suggests that expert stakeholders call for involving adolescents [31–33], and that members of the public even call for involving younger children (of course, in a way that is appropriate for their age and developmental abilities [34]) in HSV tasks [17, 35]. While the direct involvement of children in HSV tasks can and should be explored further, exploring alternative, broader approaches to their involvement in the HSV process is also warranted. Indeed, if the voices and preferences of children are deemed important, we should move beyond ongoing discussions focussing on HSV tasks and focus on exploring meaningful ways to achieve this.

## Public involvement in decisions on health(care)

The relevance of the involvement of members of the public in decisions on health(care) has long and widely been debated and researched [36–42]. According to the World Health Organization (WHO) ‘people have a right and duty to participate individually and collectively in the planning and implementation of their health care’ [43]. In 2021, the WHO published a guide under the adage *“Nothing about us*,* without us”* for involving children in decision-making [44], further expanding their methods of involving members of the public to also include children.

The arguments for public involvement in decisions on health(care) are extensive, considering that this can serve many aims, be implemented on different levels of decision-making and in various ways, ranging from members of the public being involved as mere consultants to policymakers, to them having actual decision power [38, 39, 45, 46]. In what follows below, we discuss (a non-exhaustive list of) 13 arguments for public involvement in decisions on health(care) that we identified in the broader health economics literature (i.e., not specific to HSV), and examine their relevance to involving children in the HSV process. The arguments were extracted from different sources, including academic papers and policy documents, which were identified through search engines and snowballing. We extracted arguments supporting public involvement in health(care), which, after categorizing them, resulted in the 13 arguments listed in Table 1. Note that we classified the arguments into input, process, and outcome arguments to reflect the differences in their underlying focus.

**Table 1:** Arguments for public involvement in decisions on health(care)

Classification	#	Members of the public should be involved, because…		
*Input*	(1)	public involvement is a good in itself		
	(2)	public involvement is fitting in a democratic society		
	(3)	consumers of healthcare should have a say in decisions concerning their health		
	(4)	taxpayers as financers of healthcare should have a say in decisions concerning population health		
*Process*	(5)	public involvement can improve the understanding of (constraints on) policy of those involved		
	(6)	public involvement can increase the general acceptance of (changes in) policy decisions		
	(7)	public involvement can increase the confidence for broader (political) engagement of those involved		
	(8)	public involvement can increase the understanding of individual patient’s condition and care needs		
*Outcome*	(9)	public involvement leads to the introduction of new and relevant expertise into policy		
	(10)	public involvement increases the legitimacy of the process and outcomes of decision-making		
	(11)	public involvement increases the quality of decisions by ensuring better alignment with values and priorities of the general public		
	(12)	public involvement is the best method to achieve optimal decision outcomes for society		
	(13)	public involvement can enhance a healthcare system’s responsiveness		

Arguments 1 through 4 are classified as input arguments, which describe the normative principles underlying public involvement. Arguments 1 to 3 successively describe how public involvement is considered ‘a good in itself’ (i.e., something that holds intrinsic value), how this is ‘fitting in a democratic society’ since it allows for the voices and preferences of members of the public to be heard, and how healthcare consumers should have a say in decisions that concern their own health [40, 41, 47, 48]. Argument 4 describes a widely taken perspective on public involvement in decisions on health(care) (including HSV); taxpayers as financers of healthcare should have a say in decisions concerning population health [6].

Arguments 5 to 8 are classified as process arguments, which describe the beneficial consequences of public involvement to those involved. Arguments 5 and 6 describe political and educative effects on those involved, such as how public involvement can improve the understanding of (constraints on) policy, and how it may increase the acceptance of (changes in) policy decisions [40, 41, 48]. Arguments 7 and 8 describe how being involved can increase one’s confidence for (political) engagement (also in other sectors/circumstances) and can improve understanding of patients’ conditions and care needs in the case of involvement at the service-level [40, 41].

Arguments 9 to 13 are classified as outcome arguments, which describe the beneficial consequences of public involvement on the process and outcome of decisions. Argument 9 describes that public involvement leads to the introduction of new and relevant expertise (e.g., by means of lived experiences of those involved [3, 37]) into policy [40, 41]. Argument 10 describes how public involvement can increase the legitimacy of the process and outcomes of decision-making [41], while argument 11 describes how public involvement improves the quality of decisions by ensuring better alignment with the values and priorities of the public [48]. Argument 12 describes that involving members of the public in decision-making is the best method to achieve the optimal decision outcome for society [49, 50]. The final argument, argument 13, describes that a healthcare system’s responsiveness (e.g., to the needs of healthcare consumers) can increase through public involvement [41].

Before turning to the applicability of these arguments in the context of valuing child HRQoL, we want to emphasize that arguments 11 and 12 can be seen as being closely related to the welfare economic context and background of CUAs, ultimately aimed at maximizing overall societal welfare (somehow comprising individual welfares, including health) [51, 52]. Without adequate assessment of health and welfare changes in those actually affected, decisions may not optimally contribute to the overall aim of improving societal welfare (and its fair distribution). While this further emphasizes the importance of understanding and including the preferences of those affected, including children in HSV, broader arguments for involving children in the full HSV process clearly exist as well.

## Arguments for involving children in health state valuation

Table 2 presents an overview of the 13 arguments, as well as the underlying rationale behind the relevance of each argument based on a critical assessment of whether the argument could reasonably and meaningfully apply to the context of involving children in HSV. Note that the argumentation in Table 2 is not in any way meant to be exhaustive nor did we distinguish between children of different ages.

**Table 2:** Applicability of arguments for public involvement to involving children in the HSV process

Argument for public involvement	Applicability
*(1) Public involvement is a good in itself*	If public involvement is considered to have intrinsic value (i.e., to be a good in itself), this argument likely holds irrespective of age, including in the context of HSV.
*(2) Public involvement is fitting in a democratic society*	Public involvement being fitting in a democratic society can be understood in a narrower sense (e.g., in relation to voting age) or more broadly as having a say in matters that directly or indirectly concern an individual. Under the former interpretation, children of certain ages may be excluded (as they may not have voting rights). Under the broader interpretation, they should be involved when decisions affect them, which would also apply to HSV. Note that the term ‘democratic society’ typically relates to more than voting rights solely [53].
*(3) Consumers of healthcare should have a say in decisions concerning their health*	The perceived need for consumers to have a say in decisions affecting them would logically also extend to children whenever they are ‘consumers of care’, either directly or indirectly. The HSV process may influence the (type of) care received, both now and in the future, including for children.
*(4) Taxpayers as financers of healthcare should have a say in decisions concerning population health*	This argument may not directly justify children’s involvement in HSV, as they typically do not pay taxes that directly fund the healthcare system. Nevertheless, in countries in which children are legally allowed to work and earn an income from a relatively young age, they may pay such taxes. In addition, they may purchase goods and services that are subject to indirect taxes (i.e., VAT) which may also be used to fund the healthcare system. This weakens the argument to exclude children for this reason from the HSV process. Furthermore, for decisions that impact both their future health (and hence possibility to be productive) and financial burdens, one might view children as ‘future taxpayers’, making their perspectives relevant. Finally, current taxpayers may wish to include (the preferences of) children in the HSV process.
*(5) Public involvement can improve the understanding of (constraints on) policy of those involved*	Children may learn about policy constraints from being involved in the HSV process, depending on the type of information provided and the method of involvement. The type of learnings and constraints will be context dependent.
*(6) Public involvement can increase the general acceptance of (changes in) policy decisions*	Being involved in some way in (elements of) a decision-making process may increase acceptance of policy outcomes. In principle, this applies to both adults and children alike in the context of HSV. In addition, involving children may also increase adults’ acceptance of decisions affecting child health.
*(7) Public involvement can increase confidence for broader (political) engagement of those involved*	Like for adults, being involved in the process of decision-making can increase children’s confidence for broader engagement, related to healthcare or other aspects of society (e.g., political, environmental, or social issues).
*(8) Public involvement can increase the understanding of individual patient’s condition and care needs*	The argument that public involvement enhances the understanding of an individual patient’s condition also applies to children in the context of HSV, as it may increase their understanding of (own) health and healthcare.
*(9) Public involvement leads to the introduction of new and relevant expertise into policy*	Just as with adults, children’s involvement in the HSV process may lead to the introduction of new and relevant expertise into decision-making. They can introduce lived experience on being (in ill health as) a child, offering unique insights that may otherwise be overlooked. This might include knowledge about children’s resilience, adaptability, and optimism in relation to less than perfect health states.
*(10) Public involvement increases the legitimacy of the process and outcomes of decision-making*	Public involvement is argued to increase the legitimacy of the decision-making process and its outcomes by allowing those affected to have a say. Similarly, it can be argued that the alignment of HRQoL values with children’s preferences can increase the legitimacy of decisions that affect them.
*(11) Public involvement increased the quality of decisions by ensuring better alignment with values and priorities of the general public*	Public involvement is argued to increase the quality of decisions, as they are better aligned with public values and preferences. Similarly, it can be argued that alignment of decisions with children’s preferences increases the quality of decisions that affect them, as it potentially increases the accuracy and validity of child HRQoL values. This does require sufficient confidence in both the methods used to derive preferences as well as (paternalistically) in the ‘quality of preferences’ of children (e.g., regarding how school activities or future health are valued).
*(12) Public involvement is the best method to achieve optimal decision outcomes for society*	The argument that public involvement is the best method to achieve optimal societal decision outcomes can be used to argue for children’s involvement in the HSV process. That is, involving children could aid in maximizing their welfare, which in turn contributes to better overall outcomes for society. Decisions may better represent what children find important, rather than decisions being based on assumptions made by adults. This crucially depends on methods to reliably elicit their preferences.
*(13) Public involvement can enhance a healthcare system’s responsiveness*	Insofar as HRQoL values contribute to healthcare decision-making and health system responsiveness, having value sets that adequately reflect preferences of (and/or are supported by) children themselves improves the system’s ability to respond effectively (e.g., by reimbursing interventions that contribute most to their quality of life).

*HRQoL*,* health-related quality of life; HSV*,* health state valuation*.

The (non-exhaustive) reasoning in Table 2 shows that most arguments in favour of public involvement in decision making are also relevant for involving children in the HSV process. We do note that in some cases their relevance may depend on the age of the children involved. Input arguments 1 to 3 seem to be applicable to children’s involvement in the HSV process. Indeed, in many democratic countries, policymakers actively seek to involve younger children in decisions on health(care) [18–23]. Perhaps the taxpayer argument (argument 4) is least applicable to children, but even that argument may be used in favour of involving children, e.g., as current [54] or future taxpayers. Moreover, current adult taxpayers seem to wish to include children’s preferences, also regarding HRQoL [17, 35] as they question their moral right to state preferences on behalf of children and believe involving children would enable them to make better informed decisions themselves [35].

Process arguments 5 through 8 can also be reasonably and meaningfully applied to involving children in the HSV process. Like adults, children can learn something from being involved in the HSV process. While the learning effect will likely depend on how children are involved, and the information provided to facilitate their involvement, being involved in the process of valuing child HRQoL may contribute to their development in several ways. For example, it may enhance their decision-making skills, strengthen their critical-thinking abilities, and abilities to weigh the needs and interests of different stakeholders. Furthermore, involving children may increase their (as well as adults’) acceptance of the outcomes of policy decisions. However, as only a small number of children are likely to be directly involved in HSV, the broader impact of such a learning effect may be relatively limited.

Finally, arguments 9 through 13 can also be used to argue in favour of involving children in the HSV process. Their involvement may bring new (lived) expertise to the table that is unique to their perspective and cannot be introduced by adults [31]. Involving children reduces the risk that this expertise is overlooked and, as such, improves the quality of decisions, as well as, depending on the way in which they are involved and which information they provide, the validity and accuracy of child HRQoL values. Involvement of children in the HSV process may also increase the legitimacy of decisions, as it ensures that relevant stakeholders are more comprehensively are represented and involved in the process. Highly relevant in the context of CUAs, involving children in the HSV process may result in decisions that optimally align with their preferences and hence are welfare improving. Obviously, this again depends on how children are involved, on the quality of the methods to derive preferences, and on the extent to which their preferences are recognised and used to inform the HSV process and, ultimately, decisions. Importantly, it requires several normative and empirical considerations regarding whether the preferences of children—even when elicited in (adjusted) HSV tasks—should be incorporated, the extent to which they are well-informed, and how they can be elicited accurately.

Given the relevance of these 13 broader arguments used to advocate for public involvement in decisions on health(care) and their applicability to the context of involving children in the HSV process, we believe that exploring *how* to achieve their involvement is warranted.

## Nothing about us without us

The objective of CUAs is to inform a decision-making process that is aimed at optimising health and welfare in society. When health interventions are aimed at children, it is important to understand how these interventions affect their HRQoL. Ideally, this requires involving children in the process of valuing child HRQoL in some form, rather than relying solely on adults’ views and assessment of children’s experienced health and well-being by focusing on population preferences–which, as Hausman rightly states, are not necessarily ‘good evidence of value’ [34]. Involving children in the HSV process aligns with arguments in favour of public involvement in decisions on health(care) that were drawn from the broader health economics literature. Given the concerns regarding directly involving children in HSV tasks discussed in the HSV literature (e.g [4]), the question of *how* to involve children remains crucial. In that context, broader arguments show the intrinsic value of their involvement above and beyond direct preference elicitation, contributing to the legitimacy of ultimate decisions made by policymakers.

Although the (relevance of some of the) arguments may vary with the age of the children involved, overall, the arguments discussed here, provide a basis *for* exploring the involvement of children in the HSV process. This also seems aligned with the views of important stakeholders (e.g., [31, 35]). On the one hand, if the underlying principles of the decision-making process (i.e., relating to the input and process arguments) are deemed important, children’s preferences and opinions can be considered important on the basis of their right to have a say in matters that concern them or learn from their involvement. On the other hand, if the objective is to ensure accuracy, transparency, and validity of the child HRQoL values obtained from HSV (i.e., relating to the outcome arguments), which is a core requirement for use in CUAs, involving children can help increase alignment of these values with their preferences.

Rather than aiming for conventional methods like TTOs, involving children can be achieved using more abstract, qualitative approaches (e.g., deliberative groups, as suggested by Hausman [34], even about the choice *who* to ask to perform HSV tasks on their behalf, if not themselves). While such approaches are not without their own challenges and questions (including ethical concerns, cognitive demands, and difficulties reaching consensus on the value of child HRQoL), this could be explored further. Nonetheless, a successful method built upon children’s involvement might still require some quantitative approaches. These approaches can avoid concerns raised in the HSV literature [4], like trade-offs avoiding dead or very severe health states, for example, by only ranking some health states or domains or assessing (‘validating’) preferences for EQ-5D-Y health states elicited from adults (e.g [55]). These approaches may not fully qualify as strict preference elicitation but can still provide useful information increasing both insight into the extent to which preferences elicited from adults are representative of children’s preferences, as well as the acceptability of derived HRQoL values. Furthermore, having knowledge of children’s preferences not only improves understanding of potential differences from and biases in adults’ preferences, but also ensures more informed decision-making. This may also provide useful information on the potential reasons for overruling certain preferences of children on paternalistic grounds, if deemed to be in the best (long-term) interests of the children involved. However, in such cases, it is important to understand and justify the extent to which their preferences are (not) considered and to be able to decide on the best method for refining or adjusting them. Consequently, methods to obtain these insights could and as we argue here should be explored further. In that context, it is worth noting that also the extent to which conventional HSV approaches (like TTOs) are suitable for use in different age groups of children needs further investigation. For instance, it is unclear whether modifications are needed for older children (e.g., 16 to 17-year-olds). In general, the methods used in involving children (eliciting preferences or otherwise) may differ between age groups.

Concluding, in this paper we argued that in moving forward different forms of and methods for involving children in the broad process of HSV can and should be explored further to increase the legitimacy and accuracy of child HRQoL values used in CUAs. Considering that the discussions on involving children in HSV have largely taken place among scholars, the perspective of other relevant stakeholders—including policymakers, experts, and children—are also important to explore. Even if involving children in HSV *tasks* is still considered undesirable (for whatever reason), we believe that completely excluding children from the full HSV *process* is hard to justify. It is neither aligned with broader arguments for public involvement in decisions on health(care) nor with current trends in healthcare policy making that seek to follow the adage “*Nothing about us*,* without us”*.
